# Evaluation of Sports Medicine Fellowships in the United States Based on Academic Productivity

**DOI:** 10.5435/JAAOSGlobal-D-21-00165

**Published:** 2021-10-05

**Authors:** Cory K. Mayfield, Ioanna K. Bolia, Hansel Ihn, Aryan Haratian, Laith K. Hasan, George F. Hatch, Frank A. Petrigliano, Alexander E. Weber

**Affiliations:** From the USC Epstein Family Center for Sports Medicine at Keck Medicine of USC, Los Angeles, CA.

## Abstract

Institutional academic productivity remains an influential factor in an applicant's selection of fellowship training. This study aimed to determine the quality and quantity of research in the United States orthopaedic sports medicine fellowship programs and identify those with highest productivity.

**Methods:**

The Arthroscopy Association of North America Fellowship Directory was used to evaluate 88 fellowships in the United States. Publication data and Hirschberg indices (h-index) were collected from the Scopus database. Subanalysis was performed based on the number of publications and mean h-index.

**Results:**

Total number of publications per faculty member ranged from 0 to 866, with a median of 20. The median h-index per faculty member was 9. The number of fellows was correlated with a higher mean average h-index of faculty members (*P* = 0.05). The five programs with the highest number of publications included Hospital for Special Surgery, Rush University, University of Pittsburgh Medical Center, Mayo Clinic in Rochester, and Boston Children's Hospital.

**Conclusions:**

Most academic productivity in sports medicine is produced by a relatively small number of fellowship programs in the United States. Of interest, the number of fellows or faculty does not affect significantly the quality or quantity of research productivity at top institutions.

Traditionally, orthopaedic surgery residency in the United States has consisted of 5 years of training geared toward preparing trainees for general orthopaedic practice. However, increased work-hour restrictions and supervision requirements have altered the clinical experience of orthopaedic surgery residency.^1^ As the field of orthopaedic surgery becomes increasingly subspecialized, the demand for residents to pursue fellowship training has concurrently grown to capture the increasing breadth of knowledge and technical innovations required for individual practice.^2-4^ These factors have contributed to a continued increase in pursuit of fellowship training with approximately 90% of current orthopaedic surgery trainees planning to complete at least one fellowship.^3^

Deciding on where to complete fellowship training involves factors including surgical experience, academic productivity, location, program reputation, and potential career opportunities. Although financial effects of completing fellowship training have been well documented within the orthopaedic literature, many of these factors are not readily quantifiable.^5,6^ Publicly available rankings for orthopedic hospitals exist through agencies such as US News & World Report, but these rankings include a combination of objective measures (ie, grant funding, peer-reviewed publications, and clinical trials) and subjective measures of reputation score based on physician survey results.^7^ These measures may not apply specifically to fellowship programs that may have vastly different desirability.

Academic experience available to fellows in training can be measured using several metrics. Aside from the total number of publications, Hirschberg indices (h-index) is widely used and validated to capture both the number and impact of publications produced by authors and being predictive for future scientific achievement in different medical specialties.^8-10^

This study aimed to use both publication data and h-index to determine the quality and quantity of research among faculty members within orthopaedic sports medicine fellowship programs in the United States. Quantifying this academic achievement and impact allows for applicants to decide which programs align with their overall career goals.

## Methods

### Data Collection

The Arthroscopy Association of North America (AANA) Fellowship Directory was searched (May 1, 2020) to identify orthopaedic sports medicine fellowships available in the United States.^11^ Programs were included if they had an active website with listed faculty members. Programs were excluded if they no longer had an active fellowship based on their program website or if the faculty members were not listed. The program websites were screened by three authors (I.B., H.I., and C.M.) to assure the program was an active fellowship and to determine sports medicine faculty and the number of fellows accepted each year. We further evaluated the eligible websites to identify full-time sports medicine faculty and exclude pediatric and shoulder/elbow surgeons from the final analysis.

The Scopus database (Elsevier BV), which includes research published from 1995 and, later, was subsequently queried to assess faculty-specific publication data from January 1, 1995, to May 15, 2020. The Scopus database maintains the largest available repository of citation tracking for the peer-reviewed articles. The Scopus was further used to obtain the cumulative h-index for each author over the study period. The h-index is a metric used to evaluate the impact of an author and is typically calculated based the highest number of publications for which an author has an equal or greater number of citations by other authors, with scores >20 considered to be great.^8,12^ To ensure accurate count of publications for each department, duplicate publications counted for multiple faculty members within a single department were excluded from the overall total for each department. Subanalysis was performed on the top 10 programs based on the number of publications and mean h-index. Additional analysis was performed to assess the effect of total number of publications within each department by grouping based on total publications, including <50, 50 to 149, 150 to 299, and >300 publications. Authors with exceptionally high volumes of publication, >100 and >200 individual publications, were separately investigated to determine the effects of high-volume publication on h-index.

### Statistical Analysis

Data were pooled and assessed using Microsoft Excel 2016 (Microsoft Corporation). Statistical analyses were performed using Stata IC 16 (StataCorp LLC) with continuous data evaluated using two-tailed two-sample unequal variances *t*-tests (significant set at *P* = 0.05). Multivariate logistic regressions were performed to evaluate the complete set of programs and the top 10 programs based on number of total publications to identify the effect of faculty size and number of fellows on total number of publications and mean h-index. Further multivariate logistic regression was performed to identify the effect of high-volume publications by authors on h-index.

## Results

### Faculty-specific Measures

A total of 94 programs were identified on the AANA Fellowship Directory. After screening of program websites, six programs were excluded from analysis because they were no longer active fellowship programs. Eighty-eight total sports medicine fellowships were evaluated with 689 associated faculty members. Within the period available on the Scopus database (1995 to 2020), a total of 30,210 publications were identified after removal of duplicates within each individual department. The total number of publications per faculty member over the 25-year period ranged from 0 to 866, with a median of 20. The median h-index of faculty members was 9 (range 0 to 106).

### Program-specific Measures

Within the 88 fellowship programs examined, a total of 222 fellowship positions were identified. The average number of fellows per program was 3 (range 1 to 9), with an average faculty-to-fellow ratio of 3.5. The top 10 programs based on total number of publications and h-index were identified, and the results are listed in Table 1 and Table 2. The top 10 programs had an average of five fellows, an average faculty-to-fellow ratio of 4.75, and a mean faculty member h-index of 24.13. The five programs with the highest number of publications included: Hospital for Special Surgery, Rush University, University of Pittsburgh Medical Center, Mayo Clinic in Rochester, and Boston Children's Hospital. Programs that were in the top 10 for both total number of publications and mean h-index included Rush University, Hospital for Special Surgery, Mayo Clinic in Rochester, and the University of Virginia.

**Table 1:** Top 10 Orthopaedic Sports Medicine Fellowships Based on Number of Publications

Program	Total No. of Publications	No. of Fellows	No. of Faculty Members	Faculty:Fellow Ratio	Mean h-index
Hospital for Special Surgery Program/Cornell Medical Center Program	2869	7	36	5.1	30.4
Rush University Medical Center Program	1716	5	9	1.8	39.5
University of Pittsburgh/UPMC Medical Education Program	1253	5	12	2.4	22.2
Mayo Clinic (Rochester) Sports Medicine Fellowship Program	1100	1	6	8	33.1
Children's Hospital (Boston) Program	985	3	10	3.4	26.3
Steadman Philippon Research Institute Program	991	6	7	1.2	27.3
Fairview Southdale Hospital/MOSMI Program	821	3	42	14	4.5
Cleveland Clinic Foundation Sports Medicine Program	733	3	17	5.7	14.9
University of Virginia Program	634	3	5	1.7	28.4
Kerlan-Jobe Orthopaedic Clinic Program	590	9	23	2.6	14.6

h-index, Hirschberg indices

**Table 2:** Top 10 Orthopaedic Sports Medicine Fellowships Based on Average h-Index

Program	Mean h-index	No. of Fellows	No. of Faculty Members	Faculty:Fellow Ratio	Total No. of Publications
Rush University Medical Center	39.5	5	9	1.8	1716
Ohio State University Hospital Program	35.3	2	6	3	512
Mayo Clinic (Rochester) Sports Medicine Fellowship Program	33.1	1	8	8	1100
Cincinnati Sports Medicine & Orthopaedic Center Program	31.2	4	4	1	393
Washington University Program	30.8	Not listed	4	—	394
American Sports Medicine Institute (St. Vincent's) Program	30.5	6	4	0.7	406
Hospital for Special Surgery/Cornell Medical Center Program	30.4	7	36	5.1	2869
University of Utah Program	29.3	2	6	3	539
University of Michigan Sports Medicine and Shoulder Fellowship	29.3	2	7	3.5	803
University of Virginia Program	28.4	3	5	1.7	634

h-index, Hirschberg indices

Among the top 10 programs, the number of fellows and faculty per program had no effect on both publication (*P* = 0.42 and 0.36) and h-index (*P* = 0.86 and *P* = 0.06) of these top 10 performing programs. However, when analyzing across all programs, including those outside of the top 10, both the number of fellows and faculty members had a positive correlation with total number of publications (*P* < 0.001 and *P* < 0.001). Increased number of fellows was correlated with a higher mean average h-index (*P* = 0.05). The higher number of faculty was not associated with a change in the average h-index (*P* = 0.606).

### Effect of Publication Volume

Programs were grouped based on total publications including <50, 50 to 149, 150 to 299, and >300 publications. The total number of sports medicine faculty present at each institution increased in each consecutive group from a mean of 5 (range 1 to 11), 5.5 (range 1 to 11), 8 (range 4 to 16), and 10 (range 4 to 42), respectively (*P* = 0.012). No significant difference was found in the number of fellows per year or faculty to fellow ratio in programs within high-volume publication cohorts (*P* = 0.4101 and *P* = 0.1098, respectively). The mean h-index of these cohorts increased as total number of publications of the program increased with a mean of 5 (range 1 to 9), 10 (range 3 to 20), 15 (range 6 to 23), and 21 (range 5 to 40) respectively, suggesting higher volume of publications is sufficient to increase h-index (*P* = 0.0048). When analyzing faculty with exceptionally high volumes of publications, including >100 and >200 individual articles, a strongly positive correlation was associated with increasing number of publications and h-index (*P* < 0.0001).

## Discussion

This study investigated the academic productivity of orthopaedic sports medicine fellowships across the United States. Our overall assessment demonstrated that most academic productivity is attributed to a relatively small number of fellowship programs and attendings at a national level. The number of fellows or faculty did not affect significantly the quality or quantity of research productivity at the top programs. In addition, it seemed that a higher volume of publications was sufficient to raise the h-index. There were only a few numbers of select programs that rank among the top 10 for both total publications and mean h-index.

Because prospective fellowship applicants look to evaluate programs, many methods for program assessment are inherently subjective and potentially controversial. This is evident in popular rankings, such as US News & World Report or Doximity, in which criteria are not well defined or tailored specifically for fellowship training.^7,13^ Thus, evaluating the academic productivity of clinical faculty using publication data and h-index has continued to gain popularity as an objective measure of program quality both in orthopaedics and in various surgical subspecialties.^14-17^

Although total publications capture academic productivity, it does not capture the effect that these publications have. In this context, h-indices have become an increasingly relied measure of academic productivity.^10^ This is primarily due to the ability of h-indices to the incorporate citations in addition to publication number and having a predictive value for future academic success.^8,9^ However, the findings of our analysis suggest a strongly positive correlation between increasing publication numbers and h-index (*P* = 0.004). This finding was further supported by our regression analysis of faculty with significantly high-volume publication numbers demonstrating a positive correlation with h-index. Despite these findings, h-index remains a widely used key metric within orthopaedics and across medicine to evaluate academic productivity.

In our study, the mean h-index among all sports medicine faculties was 15.02. However, this number was significantly higher when calculated across the top 10 programs, with a mean h-index of 24.13. Our findings indicate that although the academic productivity of orthopaedic sports medicine fellowship faculty members varies widely, the overall h-index compares favorably with other surgical subspecialties within orthopaedic surgery. Amongst adult reconstruction surgeons, Khan et al^18^ found a mean h-index of 12.8. Similarly, academic spine surgeons had a mean h-index of 13.6 as determined by Schoelfeld et al. In a 2015 study by Lopez et al, fellowship-associated academic hand surgeons had a mean h-index of 10.2.^14,19^ Finally, in foot and ankle fellowship programs, Sherman et al^20^ found the mean h-index of faculty to be 11.9. The results of these studies suggest that the mean h-index among academic sports medicine fellowship faculty is higher on average than other orthopaedic subspecialties.

Few previous studies have created objective lists of sports medicine fellowship program's academic productivity.^15,21,22^ In 2016, Cvetanovich et al^21^ investigated the factors associated with academic productivity and institutional academic rank among orthopaedic sports medicine fellowships. Higher h-index and years of total practice were positively correlated with academic rank within each institution. In addition, Cvetanovich et al^21^ identified regional differences in overall academic productivity of faculty. However, the authors did not rank any programs by name and instead focused their efforts on analyzing the various factors of programs and faculty members who were associated with higher h-index.^10,21^ Another study by Clark et al^23^ surveyed fellowship program directors in sports medicine based on potential variables that may influence academic productivity including amount of protected research time, number of publications per fellow, requirement to do research, and the presence of dedicated research staff to assist with projects. The authors concluded that the presence of dedicated research staff and >25 publications annually were likely associated with higher fellowship productivity; however, specific programs were not ranked or named, and the overall survey response rate was noted to be only 33% of all programs, potentially limiting the generalizability of the conclusions drawn.^23^

In our study, there were a select few programs which ranked among the top 10 in total number of publications and h-index, including Rush University, Hospital for Special Surgery, Mayo Clinic in Rochester, and the University of Virginia. To our knowledge, no previous studies ranked fellowship programs by name to allow applicants to identify the most productive academic centers. Although the abovementioned sources (Doximity and US News and World Report) have created widely popularized rankings, they have several limitations regarding orthopaedic fellowship applicants.^7,13^ These rankings include a combination of objective measures alongside subjective measures of reputation based on physician survey results. Furthermore, these rankings are nonspecific to fellowship and, instead, based on academic medical center orthopaedic departments. The results of our study provide sports medicine fellowship applicants with an objective list of programs based on academic metrics to help better inform them in their fellowship selection.

Although academic productivity is commonly included in the criteria for selection of sports medicine fellowship program by the applicants, other factors play a role toward this decision. The surgical experience and opportunity for surgical autonomy is extremely important to applicants. Other factors taken into account include sport team coverage, program location and accessibility, family-related matters such as spouse employment opportunities and childcare, social support, opportunity to attend scientific events, financial compensation, and others. A previous study surveying orthopedic residents applying to a single sports medicine fellowship program noted that the variety and complexity of surgical exposure, autonomy, and faculty reputation were the top criteria for residents in choosing where to apply for sports fellowship programs; however, it is important to note that quality or quantity of research was not included as an option in this survey.^24^ Although this study did not examine the reputation of each sports medicine fellowship program as whole, we provided an updated rank list of academic productivity (the latest report was in 2016 by Cvetanovich et al^21^) and basic program characteristics to facilitate the program selection process by applicants and potentially stimulate the programs to improve their academic records. Future studies should focus on evaluating the sports medicine fellowship programs based on balanced measures (academic productivity, surgical experience, sport coverage, quality of life, postfellowship employment opportunities, employee benefits, and others), to provide an accurate program profile to the academic community.

Our study has several limitations. The identification of programs and faculty members was based on website information, which can be incomplete or imperfect. This was evidenced by the fact that six programs listed in our initial search of the AANA registry no longer had active fellowship training programs. In addition, identifying those orthopaedic sports medicine faculty who are directly involved in training of fellows while excluding both pediatric and shoulder and elbow faculty relied on website information. We attempted to mitigate this limitation by manual screening of each individual website and faculty member profile. Although we eliminated repeat publications within departments, we did not consider author position or number of authors. This could result in repeat publications for different departments if faculty members had changed academic institutions. Furthermore, faculty listed on program websites may not accurately reflect who fellows work with in practice. A website may list more faculty than fellows work with, and consequently, this may have reduced our calculated research productivity.

This study did not account for other measures of academic productivity such as grant funding, national presentations, lectures, or visiting professorship and, instead, relied on number of publications and h-index. Although h-index is a widely used and validated measure of academic productivity, there are inherent limitations to the h-index as a completely accurate measure. As evidenced by our findings, attendings with increasing publishing volume demonstrated an associated increase in h-index on regression analysis, suggesting that high publication volume alone is sufficient to increase h-index. Furthermore, h-index is subject to false elevation in instances of self-citation, which cannot be accounted for but is believed to play a minor role in orthopaedics and its subspecialties.^25-27^ However, addition of further metrics of productivity was considered to be unnecessarily complex without providing fellowship applicants with helpful information.

## Conclusion

Most academic productivity in sports medicine is attributed to a relatively small number of fellowship programs in the United States. Of interest, the number of fellows or faculty does not affect significantly the quality or quantity of this research productivity at the top programs. Finally, only a few numbers of select programs ranked among the top 10 for both total publications and mean h-index. These results allow for applicants to identify mentors and institutions that align with their career goals to optimize academic productivity in fellowship.
